# Unmasking Diabetes in a Young Adult Male: A Case Report

**DOI:** 10.7759/cureus.88211

**Published:** 2025-07-18

**Authors:** Jimmy Joseph

**Affiliations:** 1 Internal Medicine, Aster DM Healthcare, Dubai, ARE

**Keywords:** glycated hemoglobin (hba1c), insulin, mody, monogenic diabetes, sulphonylurea

## Abstract

Maturity-onset diabetes of the young (MODY) is an underdiagnosed form of monogenic diabetes characterized by early-onset, autosomal dominant inheritance, and non-insulin-dependent hyperglycemia. This report presents the case of a 31-year-old male diagnosed with diabetes mellitus four years ago, and initially treated with biphasic insulin due to hyperglycemic symptoms, including polyuria, polydipsia, and weight loss. He had no history of diabetic ketoacidosis. Persistent hyperglycemia despite insulin therapy prompted further evaluation. A strong family history of early-onset diabetes in his father and paternal aunt raised suspicion for MODY. Laboratory investigations showed absent autoantibodies (anti-GAD, anti-ICA) and low C-peptide levels. A trial of glimepiride, a sulfonylurea, led to marked glycemic improvement, allowing complete discontinuation of insulin within six months. This clinical response supported a presumptive diagnosis of MODY, likely the HNF1A or HNF4A subtype, although genetic confirmation is pending due to cost constraints. This report underscores the importance of considering MODY in young patients with atypical diabetes presentations, especially those with a strong family history and antibody-negative, insulin-independent diabetes. Early recognition can guide appropriate therapy, reduce treatment burden, and prompt family screening.

## Introduction

Maturity-onset diabetes of the young (MODY) is a monogenic form of diabetes that arises from mutations in genes regulating pancreatic beta-cell function. First described in the 1970s, MODY represents approximately 1-2% of all diabetes cases but is frequently misdiagnosed as type 1 or type 2 diabetes due to overlapping clinical features [1,2]. MODY typically presents before the age of 25 and demonstrates an autosomal dominant inheritance pattern with non-insulin dependence [3]. Several genetic subtypes of MODY exist, with the most common being mutations in HNF1A, HNF4A, and GCK genes [4]. Diagnosis is often delayed or missed, especially in resource-limited settings where genetic testing may not be readily accessible [5]. Key distinguishing features include negative autoantibodies, preserved beta-cell function, lack of ketosis, and a strong family history of diabetes [6]. Sulfonylureas, particularly in HNF1A-related MODY, are effective and may obviate the need for insulin [7]. This report describes a case of suspected MODY in a 31-year-old male with poor glycemic control on insulin, absence of autoimmune markers, and a significant family history. A therapeutic trial of sulfonylureas yielded an excellent glycemic response, supporting the clinical diagnosis of MODY. This report highlights the diagnostic approach and therapeutic implications of recognizing MODY in young patients with atypical diabetes.

## Case presentation

A 31-year-old male presented to the outpatient department for evaluation of poorly controlled diabetes. He had been diagnosed with diabetes mellitus four years earlier at age 27, following symptoms of unintentional weight loss of 8-10 kgs, polydipsia, and polyuria. At that time, random blood sugar had been 312 mg/dL, and HbA1c had been 10.4%. He had been initiated on biphasic insulin 30/70 Mixtard (20 units pre-breakfast, 0 units pre-lunch, 16 units pre-dinner) by a general practitioner, which had moderately controlled his sugars, although never optimally. He had no past or current history of diabetic ketoacidosis. His family history was strongly positive for diabetes: his father had been diagnosed at 24 years, and his paternal aunt (elder sister of his father) had developed diabetes in her early 30s. No family members had been on insulin therapy.

**Figure 1:**
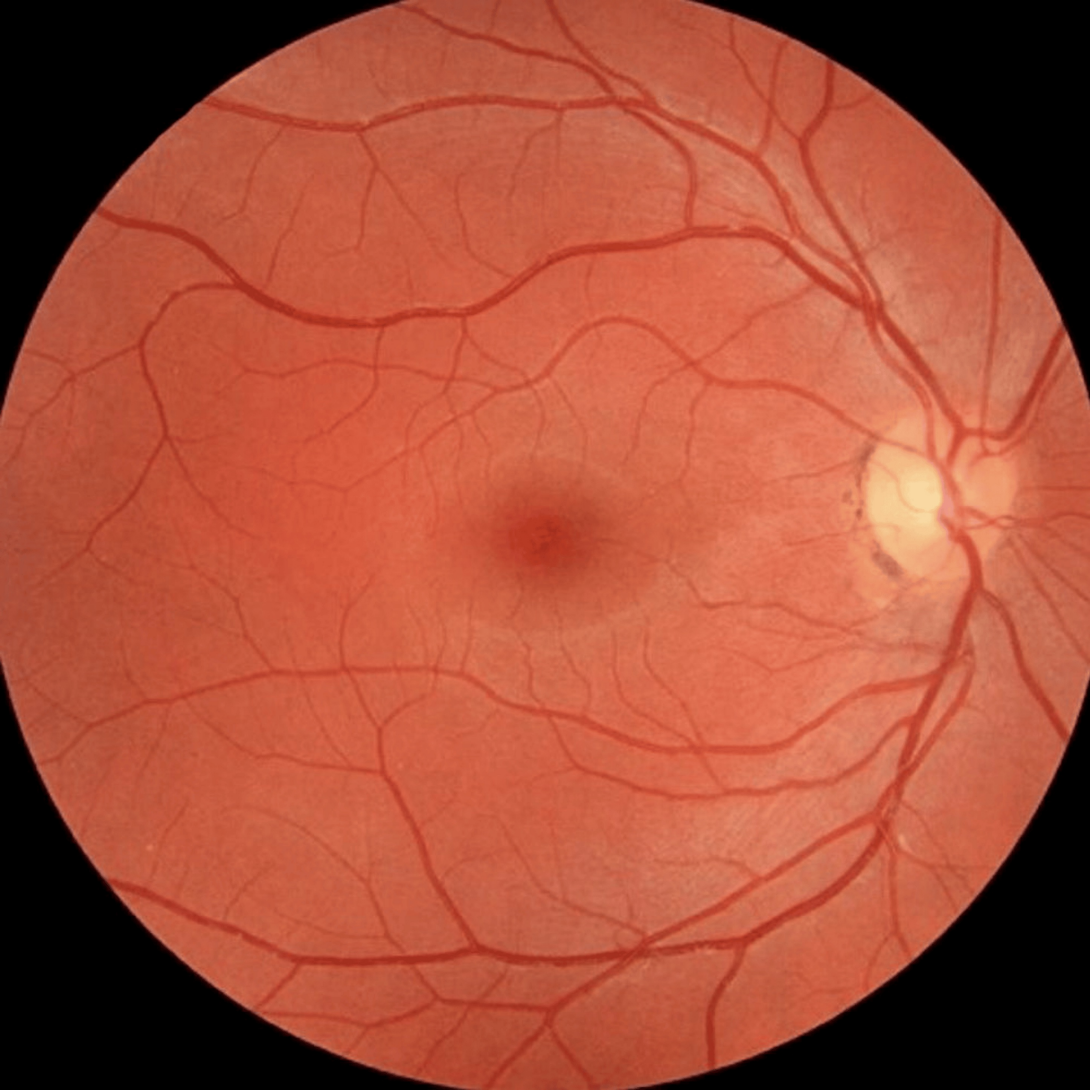
Fundus examination

The patient presented to the outpatient department with persistently elevated blood glucose levels despite adherence to insulin therapy. His clinical examination showed a BMI of 23 kg/m^2^. There was no pedal edema or signs of peripheral neuropathy. No diabetic skin changes or acanthosis nigricans were observed. Retina examination (Figure 1) revealed no evidence of diabetic retinopathy.

The laboratory investigations (Table 1) revealed a fasting blood sugar of 186 mg/dL and a postprandial blood sugar of 276 mg/dL. His HbA1c was 9.8%. Urine examination revealed albumin 1+, sugar 2+, and no ketones. Other lab parameters were within normal limits.

**Table 1:** Baseline Laboratory Investigations

Parameter	Result	Reference Range
Fasting Blood Sugar (FBS)	186 mg/dL	70–99 mg/dL
Postprandial Blood Sugar (PPBS)	276 mg/dL	<140 mg/dL
Glycated Hemoglobin (HbA1c)	9.8%	<5.7% (non-diabetic)
Serum Creatinine	0.9 mg/dL	0.7–1.2 mg/dL
C-peptide (Fasting)	0.80 ng/mL	0.81–3.85 ng/mL
Anti-GAD Antibody	Negative	Negative
Anti-Islet Cell Antibody	Negative	Negative
Urine Sugar	2+	Negative
Urine Ketones	Negative	Negative
Urine Albumin	1+	Negative

Given the early age of onset, absence of ketosis, and strong family history, autoimmune type 1 diabetes was deemed unlikely. Further evaluation revealed a fasting C-peptide level of 0.80/mL (low); anti-GAD antibody and anti-islet cell antibody were negative, consistent with a non-autoimmune form of diabetes. A clinical diagnosis of MODY was considered, likely HNF1A or HNF4A mutation. Genetic testing was advised, but the patient declined due to financial constraints. A therapeutic trial with glimepiride 2 mg twice daily was initiated, and insulin was gradually tapered over the next six months.

**Figure 2:**
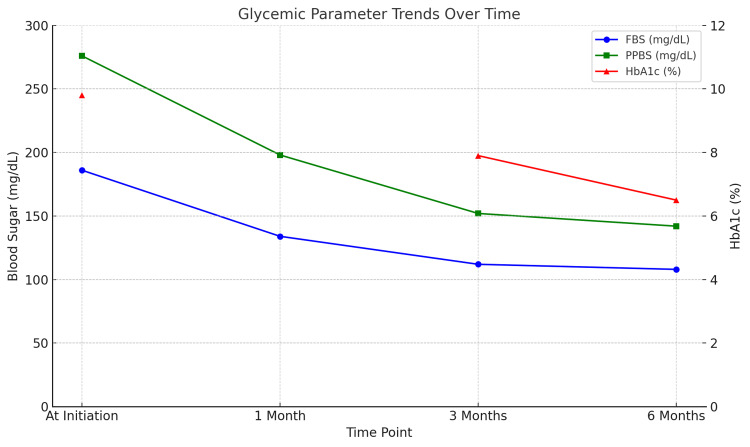
Glycemic Trend After the Initiation of Sulphonylurea FBS: fasting blood glucose; HbA1c: glycated hemoglobin; PPBS: postprandial blood sugar

Over the following six months, the patient’s glycemic control improved significantly (Table 2). His fasting and postprandial glucose readings normalized (FBS: 108 mg/dL, PPBS: 142 mg/dL), and HbA1c dropped from 9.8% to 6.5% (Figure 2). Insulin was successfully discontinued in six months without any recurrence of hyperglycemia.

**Table 2:** Glycemic Trend over Six Months FBS: fasting blood glucose; HbA1c: glycated hemoglobin; PPBS: postprandial blood sugar

Time Point	FBS (mg/dL)	PPBS (mg/dL)	HbA1c (%)
At Initiation	186	276	9.8
1 Month	134	198	-
3 Months	112	152	7.9
6 Months	108	142	6.5

The patient remains on glimepiride monotherapy with the dose reduced to 2 mg in the morning and 1 mg at night daily, with continued euglycemia at six-month follow-up. His father has been advised to be evaluated for possible MODY, given the clinical implications for family members.

## Discussion

MODY refers to a heterogeneous group of monogenic diabetes characterized by autosomal dominant inheritance and early-onset non-insulin-dependent hyperglycemia. Unlike classic type 1 or type 2 diabetes, MODY is caused by mutations in genes involved in pancreatic beta-cell development, glucose sensing, or insulin secretion [3]. Thirteen MODY subtypes have been identified so far, with HNF1A, HNF4A, and GCK mutations accounting for the majority of cases [8]. The clinical presentation can resemble either type 1 or type 2 diabetes, leading to frequent misdiagnosis. Patients with MODY may be mistakenly treated with insulin despite not requiring it, as in our case [9]. Clues pointing to MODY include the onset of diabetes before age 35, absence of obesity and insulin-resistance features, absence of diabetic ketoacidosis, negative beta-cell autoantibodies, and a strong family history consistent with autosomal dominant inheritance [6].

Our patient fulfilled nearly all these criteria. The absence of ketosis and autoantibodies, low but preserved C-peptide levels, and robust response to sulfonylurea therapy are classic features of HNF1A-related MODY [4]. The patient’s strong family history and glycemic improvement on oral therapy after cessation of insulin further support the diagnosis. Sulfonylureas are the preferred treatment for HNF1A and HNF4A MODY as these mutations impair glucose-stimulated insulin secretion but preserve sensitivity to sulfonylureas [7]. Patients often exhibit dramatic improvement with low-dose sulfonylureas, allowing insulin withdrawal, as observed here. While genetic testing is the gold standard for diagnosis, it remains expensive and inaccessible in many settings [5]. In such cases, a clinical diagnosis supplemented by therapeutic response is reasonable and can guide management [10]. Referral to specialized centers and family screening should be considered once the diagnosis is established.

The risk of diabetic complications in MODY varies by subtype. HNF1A and HNF4A MODY are associated with microvascular complications similar to type 1 and type 2 diabetes, while GCK-MODY usually has a benign course and does not require treatment [11]. Differentiating MODY from other types of diabetes has major implications; it prevents unnecessary lifelong insulin use, allows cost-effective sulfonylurea therapy, facilitates early detection and management of diabetes in family members, and enables genetic counseling [2]. In India and other developing countries, MODY is likely underrecognized due to limited awareness and access to diagnostic modalities [11]. Studies from South Asia estimate a prevalence of 2-5% among young-onset diabetes cases [12]. In this case, the clinical suspicion, strategic withdrawal of insulin, and favorable response to oral agents proved crucial. Continued education of clinicians about MODY and provision of affordable genetic services will help improve diagnosis and outcomes in these patients.

## Conclusions

This report underscores the importance of considering MODY in young adults with atypical diabetes presentations, particularly those who are antibody-negative, ketosis-free, and have a strong family history. Our patient, initially misdiagnosed and managed as a case of type 1 diabetes, was found to have features suggestive of MODY and responded favorably to sulfonylurea therapy with complete insulin discontinuation. Early and accurate diagnosis of MODY can significantly affect management by reducing unnecessary insulin use, improving glycemic control with oral agents, and guiding family screening. Although genetic testing remains the diagnostic gold standard, in settings where such testing is not feasible, a combination of clinical features, laboratory tests, and response to treatment can be highly informative. This report highlights the need for heightened clinician awareness of monogenic diabetes and supports the trial use of sulfonylureas in suspected MODY cases after excluding other diabetes types. Increased accessibility to genetic services and raising awareness on MODY will further improve detection rates and patient outcomes.
